# Deep Learning Applications in Clinical Cancer Detection: A Review of Implementation Challenges and Solutions

**DOI:** 10.1016/j.mcpdig.2025.100253

**Published:** 2025-07-18

**Authors:** Isaiah Z. Yao, Min Dong, William Y.K. Hwang

**Affiliations:** a Hwa Chong Institution, Junior College Section, Singapore; b National Cancer Centre Singapore; c Singapore General Hospital, SingHealth, Singapore

## Abstract

Deep learning (DL) has revolutionized cancer detection accuracy, speed, and accessibility. Leveraging sophisticated algorithms, DL has demonstrated transformative potential across diverse applications, including imaging-based diagnostics and genomic analysis, ultimately leading to better detection, improved patient treatment outcomes, and decreased overall mortality rates. Despite its promise, integrating DL into clinical practice presents substantial challenges, including limitations in data quality and standardization, as well as ethical and regulatory concerns, and the need for model interpretability and transparency. This review provides a comprehensive analysis of recent research (2018-2024) retrieved from PubMed and IEEE Xplore databases, encompassing 1304 studies from PubMed and 115 from IEEE, to highlight the current applications, opportunities, and challenges of DL in oncology. Additionally, this paper explores emerging solutions, including federated learning, explainable artificial intelligence, and synthetic data generation, to address these barriers. The review also emphasizes the importance of interdisciplinary collaboration, the integration of next-generation artificial intelligence techniques, and the adoption of multimodal data approaches to improve diagnostic precision and support personalized cancer treatment. By systematically analyzing key developments and challenges, this review aims to guide future research and DL technologies in oncology, promoting equitable and impactful advancements in cancer care.

**Article Highlights**

• Deep learning models have demonstrated high accuracy in detecting various cancers across modalities such as radiology, pathology, and genomics, offering promising tools for early diagnosis and prognostication.
• Despite this promise, the clinical integration of deep learning faces challenges, including data bias, lack of interpretability, regulatory hurdles, and disparities in real-world deployment across health care systems.
• Robust model validation using diverse, multi-institutional data sets and explainable artificial intelligence approaches is critical to building trust and ensuring generalizability in clinical settings.
• Future progress depends on fostering collaborative ecosystems that unite clinicians, data scientists, regulators, and patients to address ethical, technical, and practical barriers to widespread adoption.

Cancer is a leading cause of morbidity and mortality worldwide, with an estimated 19.3 million new cancer cases (18.1 million excluding nonmelanoma skin cancer) and almost 10.0 million cancer deaths (9.9 million excluding nonmelanoma skin cancer) occurring in 2020 alone.^1^ Fortunately, cancer diagnosis and treatment have evolved substantially, transitioning from rudimentary detection methods to more sophisticated imaging and molecular profiling techniques. These advancements have improved early detection and treatment precision; however, the high complexity and heterogeneity of cancer continue to demand innovative solutions.^2^

In recent years, artificial intelligence (AI) has emerged as a transformative force in health care, with deep learning (DL) at its forefront.^3^ DL is a subset of machine learning (ML) within AI,^4^ focusing on algorithms inspired by the human brain, known as artificial neural networks.^5^ DL excels at analyzing large amounts of unstructured data (such as images, text, and audio), learning patterns, and making predictions with minimal human intervention. It has enabled state-of-the-art performance in various complex tasks across industries. Neural networks consist of layers of interconnected nodes (neurons) that process data and pass it forward.^6^ A typical neural network has 3 layers: an input layer that accepts raw data; a hidden layer that transforms the data through weights, biases, and activation functions; and an output layer that provides the final prediction or classification.^7^ Simple tasks require one hidden layer, whereas complex tasks need 2 or more hidden layers, forming a deep neural network (DNN). The DNNs learn complex representations and hierarchies of data features (eg, identifying edges in an image at early layers and recognizing objects in later layers), allowing automatic feature extraction from raw data.^3^ DL has revolutionized cancer diagnostics by processing multimodal data sets including medical imaging, genomic information, and clinical records,^8^ facilitating breakthroughs in imaging-based diagnostics and genomic analysis.^9^ For instance, convolutional neural networks (CNNs) have revolutionized radiology, enabling the early detection of malignancies in computed tomography (CT) scans, mammograms, and histopathology images.^10^^,^^11^ Similarly, DL models have been pivotal in genomic analysis, identifying inconspicuous cancer-associated mutations and biomarkers^12^ to improve early detection, diagnostic accuracy, and personalized treatment plans.^13^ Despite its promise, the adoption of DL in clinical oncology faces challenges such as data scarcity, ethical and regulatory concerns, interpretability of complex models, and scalability limitations.^14^ Furthermore, the sensitive nature of patient data necessitates stringent measures to ensure privacy and security,^15^ whereas biases in training data sets could exacerbate health care disparities if left unaddressed.^14^ Addressing these challenges is critical to unlocking the full potential of DL in cancer care.

## Literature Review

We searched the PubMed and the IEEE Xplore database for articles published between 2018 and 2024 in the English language, using Boolean Logic terms like “AND” and “OR” to narrow or widen the search scope. For example, (“Deep Learning”[Title/Abstract] OR “Artificial Intelligence”[Title/Abstract]) AND “Cancer Detection”[Title/Abstract]), for the PubMed repository. Furthermore, we also used IEEE and PubMed’s wildcards, such as detect∗, for detect, detection, and detecting. The search terms used were “Deep Learning” or “Artificial Intelligence” and "Cancer Detection." Studies on cancer detection using DL were considered eligible.

The inclusion criteria were studies involving individuals older than 18 years of age, novel DL models, peer-reviewed and non-peer-reviewed publications, and cancer-related research. The number of eligible articles was drastically reduced by approximately 70% after applying these criteria.

A thorough study of the selected research papers involved answering quality control questions such as (1) whether the study covered all aspects of the review's topic, (2) the quality of the paper, and (3) the uniqueness of the results. The first question assessed the topic coverage of the 145 selected papers, yielding a 77% satisfactory result. The second question verified the quality of the papers, resulting in an 82% satisfactory result.

This process yielded 1122 studies from PubMed, of which 242 were reviews, and 2270 from the IEEE database for the query, of which 208 were reviews, with only 174 meeting the strict criteria of relevance, methodological quality, and population specificity while also avoiding studies that replicated or marginally improved upon prior work. This rigorous screening (Supplemental Appendix, available online at https://www.mcpdigitalhealth.org/) ensured that only articles with direct relevance and methodological soundness were included, enabling an in-depth exploration of core research themes, model performance benchmarks, and clinical implications.

### Current Application of DL in Cancer Detection

#### Imaging-Based Diagnostics

AI algorithms, particularly CNNs, have revolutionized early cancer detection by analyzing medical imaging data and identifying potential tumors by comparing patterns from existing data sets. These models excel in identifying early stage malignancies in radiographs, mammograms, and CT scans. For example, in lung cancer, low-dose CT imaging combined with AI enhances the detection and classification of early stage lesions, improving survival rates.^15^^,^^16^ Breast cancer screening has been enhanced through AI’s ability to detect microcalcifications and subtle architectural distortions in mammograms, achieving high sensitivity and specificity and reducing the rate of false negatives.^17^ DL models, particularly CNNs, significantly enhance radiomics by learning and extracting highly specific and complex imaging patterns, such as texture, shape, and intensity. It eliminates the need for manual feature engineering, allowing for the automatic discovery of imaging biomarkers associated with malignancy or treatment response. As such, the extraction of quantitative imaging features that provide insights beyond what is visible to human observers through radiomics can be enhanced by DL.^18^ Through DL, these features can distinguish between benign and malignant lesions, aiding in early detection and diagnosis of cancer and personalized treatment planning.^19^

CNNs excel at detecting subtle and complex imaging patterns that may not be visible to the human eye, improving early cancer detection. Furthermore, DL eliminates the need for manual feature engineering, automatically identifying important imaging biomarkers.^20^

However, CNNs require large, diverse, high-quality data sets to avoid biases. If the training data are skewed (eg, predominantly from one ethnic group), the model may perform poorly on other groups. Furthermore, without careful design and validation, models may memorize noise or specific features of the training set, resulting in poor performance on unseen data.^21^ Medical imaging protocols vary across hospitals; hence, a model trained on data from one hospital may not perform well at another hospital unless it is carefully adapted.^22^

#### Genomic and Molecular Diagnostics

DL has revolutionized cancer genomics by enhancing diagnostic accuracy and enabling personalized medicine through the development of advanced computational models. These systems integrate genomic data with other diagnostic tools, such as radiomics and pathology imaging, to create a more comprehensive framework for cancer detection, thereby improving clinical decision making. One key challenge in genomic analysis is the presence of imbalanced data sets, which can lead to biased predictions. To address this, methods like SMOTE-Tomek resampling help balance training data, making DL models more robust and generalizable across patient populations.^23^ DL models have also been employed to identify cancer-specific biomarkers, such as HE4 and CA125 for ovarian cancer and microarray gene data for leukemia, allowing for precise molecular classification of tumors.^24^ Notably, weighted CNNs have demonstrated high accuracy in leukemia prediction, showcasing the ability of AI models to process vast genomic datasets effectively.^23^ Tools like the Galleri test utilize DL to analyze circulating cell-free DNA (cfDNA) for early cancer detection, even in cases where established screening protocols are not available.^25^ Combining AI techniques, such as weighted CNNs with feature selection algorithms (eg, Chi2), has led to near-perfect accuracy (99.9%) in leukemia prediction using high-dimensional microarray gene data.^23^ These advancements underscore the potential of DL to transform cancer detection by optimizing biomarker identification, refining genomic analysis, and providing patients with more timely treatment options, ultimately contributing to better patient outcomes.

These AI systems not only detect a cancer signal but also predict where the cancer is originating with high accuracy (∼88.7%), guiding clinical follow-up and reducing unnecessary tests.^26^ Furthermore, weighted CNNs, combined with feature selection (Chi2), can handle tens of thousands of genes, effectively reducing noise and focusing only on important markers. This enables extremely high diagnostic precision (>99% accuracy), even in highly complex datasets such as microarrays.

However, although tests like Galleri show excellent performance in advanced cancers, sensitivity is significantly lower in stage I cancers (∼16.8%), limiting impact in truly “early” detection. Also, detecting very early tumors or indolent cancers may lead to unnecessary biopsies or overtreatment, especially if the detected cancer would not have become clinically significant.^26^

#### Multimodal Data Integration

Multimodal data integration is a critical approach in DL applications for cancer detection,^23^ leveraging various types of data to improve diagnostic accuracy and prognostic capabilities. Integrating imaging, clinical, and genomic data using DL offers a holistic approach to cancer detection. Studies combining biomarkers such as HE4 and CA125 with imaging data achieved over 86% diagnostic accuracy for predicting ovarian cancer, emphasizing the importance of multimodal analysis.^27^ DL models analyze genomic mutations and patterns in conjunction with imaging data (eg, radiomics or magnetic resonance imagining [MRI]/CT scans) to refine tumor classification and enhance early detection. For instance, radiogenomic models predict tumor biology from imaging phenotypes,^28^ and the combination of cfDNA analysis with imaging features aids in detecting hidden tumors.^29^ DL models can also align microscopic pathology data with macroscopic imaging data to make more accurate predictions.^30^ Through multimodal data integration, models that combine genomic markers (eg, BRCA mutations) with serum biomarkers (eg, CA125 for ovarian cancer) exhibit improved predictive performance.^31^ For example, multicancer early detection (MCED) tests integrate circulating tumor DNA with methylation profiles for broad cancer detection,^32^ and ovarian cancer prediction models integrate HE4 and CA125 serum data with genomic insights.^33^

cfDNA analysis detects tumors too small to be seen on imaging; when combined with radiomics, early stage cancers can be identified more reliably, allowing for better detection of small or hidden tumors.^30^ Furthermore, by combining imaging (MRI and CT), serum biomarkers (HE4 and CA125), and genomic data (eg, BRCA mutations and cfDNA), models gain a fuller biological picture of tumors, improving diagnostic accuracy.^28^

However, different modalities (eg, cfDNA, MRI, and genomics) have distinct data structures (sequences, images, and tabular), making it technically challenging to align and integrate them properly.^27^ Most data sets also focus on imaging or genomics alone; very few public data sets have synchronized imaging, cfDNA, and clinical data for the same patients.^31^ Finally, the more complex and multimodal a model becomes, the harder it is for doctors to understand why it made a certain cancer prediction, which may limit clinical adoption.^27^ Table 1 summarizes the current applications of DL in cancer detection.

**Table 1:** Applications of DL in Cancer Detection

Application	Key points
Imaging-based diagnostics	- CNNs identify patterns in imaging data (e.g., CT scans, mammograms). - Improved sensitivity and specificity in detecting malignancies (e.g., lung and breast cancer). - Radiomics leverages DL to identify complex imaging biomarkers. - High resolution detection capabilities - Need large datasets to train
Genomic and molecular diagnostics	- DL integrates genomic and diagnostic data, overcoming data imbalances using techniques like SMOTE-Tomek. - Tools like Galleri analyze cfDNA for early cancer detection. - High accuracy (e.g., 99.9% in leukemia prediction) with advanced models. - High diagnostic precision - limited early detection capabilities
Multimodal data integration	- Combines imaging, clinical, and genomic data for holistic diagnostics. - Example: Multicancer early detection tests integrate cfDNA with methylation profiles. - High resolution detection capabilities - Faced difficulties when faced with large datasets

### Current Opportunities of DL in Cancer Detection

#### Improved Diagnostic Precision

Opportunities for improving diagnostic precision through DL in cancer detection are abundant, particularly when leveraging advanced computational models and diverse data modalities.^34^ DL models excel in identifying subtle patterns in complex data sets, improving early detection rates. For instance, blood-based MCED tests utilize DL to analyze cell-free DNA, achieving a 97% accuracy rate in predicting the origins of cancer signals.^28^ Integrating genomic data (eg, mutations and methylation patterns) with radiomics features enables models to link molecular signatures with visible tumor characteristics, refining tumor classification and progression predictions.^35^ Combining histopathological images with imaging modalities like MRI or CT enhances detection accuracy, particularly in cancers with heterogeneous presentations, such as glioblastomas.^36^ CNNs excel at analyzing imaging data, detecting subtle features that may be imperceptible to radiologists, such as microcalcifications in breast cancer^37^ or early stage lung nodules.^38^

#### Integration of Multimodal Data

The integration of diverse data types enables DL to provide comprehensive diagnostic insights, including multimodal data integration. Multimodal systems enhance prognostic predictions and facilitate personalized treatment planning by simultaneously analyzing imaging, genetic, and clinical data.^39^ By combining radiological images (CT/MRI) with genomic markers (eg, cfDNA mutations and transcriptomics), DL models can extract complementary features, improving tumor detection and classification.^40^ Multimodal data integration enables precision cancer diagnostics by aligning genomic mutations (eg, BRCA1/BRCA2), imaging features (eg, tumor size and margin irregularity), and serum biomarkers (eg, HE4 and CA125 for ovarian cancer). This stratification ensures tailored screening and treatment plans based on a patient’s risk profile.^29^

#### Pathology in Cancer Detection

Pathology provides definitive answers for cancer detection through the combined use of histopathology, cytopathology, and molecular pathology. Currently, DL models, such as ResNet50 and Hover-net, are being utilized to classify histopathological images of breast cancer.^41^ Another area that can be developed is in whole slide images (WSIs).^42^ WSIs are enormous in size (often several gigabytes) because they capture a detailed view of the entire specimen.^43^ CNNs can analyze WSIs by breaking them into smaller patches. These patches are classified as normal or cancerous based on the features they have been trained to recognize.^44^ Other AI models, such as the GPT model, can create graph-based representations using vision transformers to improve WSI classification accuracy, in this case with the GPTs, distinguishing between adenocarcinoma and squamous cell carcinoma.^45^ These advancements can actually solve the problem of some rare cancers with deep-infiltrating cells, which are difficult to separate from healthy tissue. Through CNNs breaking WSIs into smaller patches, the CNNs can accurately detect whether a cell is cancerous on a cellular level. However, this can lead to higher processing times as the CNN must focus in greater detail on more images (a smaller area of focus will result in more images that need to be analyzed).

Researchers typically utilize the Cancer Genome Atlas (TCGA) to obtain H&E-stained WSIs across 13 tumor types, focusing on tumor-infiltrating lymphocytes (TILs),^46^ and also use the molecular and Cellular Oncology Colorectal cancer study WSI data set for digitized tissue slides related to colorectal cancer.^47^ These repositories provide high-quality, annotated data sets, which are essential for training DL models. These data sets also encompass data from all over the world, thereby reducing the likelihood of imaging bias toward specific groups and populations.

For TCGA repository, researchers will utilize CNNs to analyze H&E-stained WSIs. The CNNs were trained on small image patches extracted from WSIs to classify regions based on the presence or absence of TILs. Customized architectures were then employed to create TIL density maps across entire slides. The resulting TIL maps provided a quantitative spatial distribution of immune cells within tumors.^48^

For the molecular and cellular oncology colorectal cancer repository, researchers utilized weakly supervised DL techniques, such as a multiple instance learning (MIL) framework, to predict patient survival from WSIs. The models were trained with slide-level labels instead of with detailed annotations. A higher-level MIL pooling method will result in patch-level predictions being aggregated into a slide-level prognostic prediction. This achieved good predictive accuracy for patient survival, outperforming traditional histopathological grading systems such as the simple tumor grade.^49^

Although cancer has different cancer contents, it can be easily detected through the methods mentioned above. Through WSIs and CNNs, one can easily visualize the percentage density of tumor cells in a sample and thus effectively determine the type of cancer it is.

Another added feature of this is that it can detect what type of cancer is present in the sample. For example, HALO-AI for pulmonary neuroendocrine tumors utilized a CNN-based approach to differentiate lung neuroendocrine tumors, including small cell lung carcinoma, large cell neuroendocrine carcinoma, and atypical carcinoid (AC). It achieved a mean F1 score of 0.99 on the testing set, indicating high diagnostic accuracy. small cell lung carcinomas have a cancer density of 70%-90% of tumor area, whereas large cell neuroendocrine carcinomas have a slightly lower tumor density of 60%-80% of tumor area. ACs have the lowest density, ranging from 40% to 60% of the tumor area.^50^ Table 2 summarizes the current opportunities in this field.

**Table 2:** Current Opportunities

Opportunities	Key points
Improved diagnostic precision	DL enhances early detection by analyzing subtle patterns in data (e.g., cell-free DNA in blood-based MCED tests with 97% accuracy). - Combines genomic and radiomic data for better tumor classification and progression predictions. - CNNs detect subtle imaging features like microcalcifications (breast cancer) and early lung nodules. - Integrates histopathological images with MRI/CT for improved detection of heterogeneous cancers like glioblastomas.
Integration of multimodal data	- Multimodal systems combine imaging, genetic, and clinical data for personalized treatment and better prognostic predictions. - Aligns genomic markers (e.g., BRCA mutations) with imaging features (e.g., tumor size) and biomarkers (e.g., HE4, CA125) to refine cancer diagnostics. - Enables tailored screening and treatment based on patient-specific risk profiles.
Pathology	- Models such as ResNet50 and Hover-net are used to classify histopathological images, especially for breast cancer. - WSIs are analyzed by CNNs in smaller patches to detect cancer cells at a detailed level, although this increases processing time. - Advanced models like GTP use vision transformers to improve classification. - Datasets like TCGA and the MCO CRC Study provide annotated H&E-stained WSIs for diverse cancer types, reducing bias and enabling global applicability. - Models using MIL predict patient survival from WSIs without detailed annotations, outperforming traditional grading methods. - CNNs can determine tumor cell density and distinguish between cancer types based on WSI patterns.

### Challenges of DL in Cancer Detection

#### Dataset Size Limitations

The success of DL models hinges on the availability of large, high-quality data sets. However, data scarcity, biases, and variability across populations remain significant barriers. Cancer detection often involves small data sets, especially for rare cancers, but DL models require large amounts of data to learn effectively and avoid overfitting. Furthermore, the high cost and difficulty of collecting annotated medical data, as well as privacy concerns and restricted access to clinical data sets, make it challenging to collect large amounts of data on tumors, especially rare ones.^41^ One solution is data augmentation, where techniques such as flipping, rotating, cropping, and scaling medical images artificially expand data sets to improve model training.^51^ For example, histopathology slides can be augmented to simulate variations in microscopy.^52^ Furthermore, federated learning offers a potential solution by enabling collaborative model training across institutions without data sharing.^53^ Additionally, generative adversarial networks (GANs) and variational autoencoders (VAEs) generate synthetic medical images and genomic data that resemble real-world samples. This can help augment data sets for rare tumors.^54^ However, federated learning is a relatively new concept, dating back to only 2016.^55^ Different hospitals employ different collection methods, which can hinder data collection and collation. This can be resolved by developing a standard format for data presentation, thereby minimizing and reducing differences in data quality.

#### Ethical and Regulatory Concerns

Ethical and regulatory concerns pose significant challenges, particularly due to the sensitive nature of medical data, the high stakes of cancer diagnostics, and the evolving regulatory landscape surrounding the use of AI in health care. The most important ethical concern is about patient data privacy and security. DL models require access to large-scale patient data, including imaging, genomic, and clinical records, which often contain personally identifiable information. Sharing or mishandling such data risks breaching patient privacy laws such as Health Insurance Portability and Accountability Act (HIPAA) in the US and General Data Protection Regulation (GDPR) in Europe.^56^ Unauthorized access or breaches can harm patient trust and compromise health care systems, whereas limited data sharing hinders the development of robust DL models for cancer detection. Furthermore, DL models can inadvertently perpetuate biases present in training data sets, leading to inaccurate predictions for underrepresented groups, such as racial or ethnic minorities, and socioeconomic or geographic disparities in cancer data.^46^ Biased algorithms may misdiagnose or underperform for specific populations, exacerbating existing health inequities. Current bias within the medical field, such as sparse clinical and treatment data for minority groups, leads to difficulties in implementing DL for such groups, exacerbating the issue. As such, regulatory frameworks emphasizing transparency and fairness are critical to addressing these issues,^57^ although more effort needs to be put into closing the gap in medical research and knowledge for “other” identities. Many institutions have adopted federated learning, which enables DL models to be trained across multiple institutions without sharing raw patient data, ensuring privacy compliance.^58^ Furthermore, removing or masking personally identifiable information from data sets effectively anonymizes the data, complying with regulatory standards.^59^ Medical institutions have also implemented encryption and access control protocols to protect sensitive data, such as homomorphic encryption and distributed ledger computing.^60^ To combat biases, hospitals strive to ensure that DL models are trained on datasets that reflect diverse demographics, cancer subtypes, and clinical settings. They also regularly evaluate models for biases and their impact on different population groups as part of their bias auditing process.^39^

#### Model Interpretability

Model interpretability is another significant challenge in cancer detection using DL. Although DL models, particularly DNNs, achieve high accuracy, their “black box” nature makes it difficult to explain how predictions are made.^61^ In health care, where decisions directly impact patient outcomes, interpretability is crucial for clinical trust, adoption, and accountability.^62^ DL models often provide predictions (eg, benign vs malignant) without explaining why or how they reached that decision. This may be detrimental to the cancer detection process as doctors need to understand why a specific region of an image was flagged as cancerous or not in order to provide adequate care for the specific situation.^63^ Misinterpretation or unexplained errors can lead to misdiagnosis, impacting patient safety. Furthermore, a lack of interpretability reduces confidence among patients and clinicians in AI-driven cancer diagnostics.^64^ To combat this, techniques such as the Explainable AI (XAI) techniques were developed. XAI methods aim to make DL models transparent by providing human-interpretable insights such as saliency maps and heatmaps, along with feature importance methods and attention mechanisms.^65^ This ensures that DL diagnosis aligns with clinical reasoning. Techniques like gradient-weighted class activation mapping highlight regions in medical images (eg, CT, MRI, and histopathology) that influenced predictions. For example, gradient-weighted class activation mapping can show the exact tumor region contributing to a “malignant” classification.^66^ Furthermore, tools like SHapley Additive exPlanations (SHAP) and local interpretable model-agnostic explanations (LIME) analyze the importance of input features (eg, genomic mutations and biomarker levels) in a prediction. In genomics, SHAP can identify specific mutations contributing to cancer subtype predictions.^67^ Models equipped with attention layers highlight critical areas of input data that contribute most to the decision. For cancer imaging, attention mechanisms can focus on tumor margins or texture anomalies.^68^ Despite these advancements, although XAIs enhance interpretability, they do not fully eliminate the risk of model bias or errors.^69^

#### Deployment and Scalability

Implementing DL in real-world settings poses challenges related to computational requirements, workflow integration, and model generalizability. Ensuring consistent performance across diverse clinical environments necessitates rigorous validation.^70^ Deployment and scalability are critical challenges in applying DL for cancer detection.^22^ Although DL models show promise in research settings, transitioning these models into real-world clinical practice at scale remains complex. This is because DL models require significant computational power, such as graphics processing units/tensor processing units, for both training and inference. However, many health care systems, especially in resource-limited settings, lack the necessary infrastructure to deploy DL models.^71^ High computational costs and infrastructure disparities prevent scalability across hospitals and regions, particularly in low-resource environments.^72^ This also ties into the ethical and regulatory restrictions of DL, as the gap between different groups, this time on a larger scale, will increase, and those already less advanced will only continue to be left behind. Unless policies are implemented to close this gap, the socioeconomic disparity between different communities will grow. Furthermore, integrating DL models into existing clinical systems, such as electronic health records and picture archiving and communication systems, is challenging due to differences in software formats and workflows. Poor integration leads to workflow disruptions, resistance from clinicians, and reduced adoption of DL-based tools.^73^ As such, hospitals have begun to deploy DL models via cloud platforms, facilitating access to high computational power without requiring on-site infrastructure.^74^ Other techniques, such as model pruning and quantization, optimize DL models to run efficiently on low-power devices, ensuring real-time inference with minimal resources.^75^ To ensure seamless data exchange between hospitals, many hospitals ensure that their own DICOM tools adhere to standards such as DICOM for medical imaging and Fast Healthcare Interoperability Resources (FHIR) for electronic health records integration.^76^ However, cloud-based deployment raises significant concerns regarding the security and privacy of sensitive patient data. Even with encryption protocols, the risk of data breaches or unauthorized access remains a critical issue, especially in regions with stringent data protection regulations like GDPR in Europe and HIPAA in the United States.^77^

#### Clinical Trust

Clinician trust and interpretability remain critical concerns in AI-driven cancer detection. Many oncologists and pathologists are skeptical about AI-generated insights. This skepticism stems from concerns about misinterpretation of AI predictions, where models may flag variants of unknown significance without a clear biological context, making it difficult for clinicians to determine their relevance in treatment decisions.^78^ Additionally, over-reliance on AI without proper validation can lead to false positives or misleading results, potentially influencing clinical decisions in ways that may not align with established medical guidelines.^79^

To address these concerns, XAI techniques are being developed to improve model interpretability, allowing clinicians to understand how AI arrives at its conclusions.^80^ However, widespread adoption of AI in oncology and pathology still requires rigorous validation studies, interdisciplinary collaboration, and regulatory oversight to ensure AI-generated insights are both reliable and clinically actionable. Table 3 summarizes the current challenges of DL in cancer detection.

**Table 3:** Current Challenges

Challenge	Key points
Dataset size limitations	- Small datasets, especially for rare cancers, hinder DL model training and can lead to overfitting. - High cost, annotation difficulty, and privacy concerns limit data collection. - Solutions include data augmentation, federated learning, and synthetic data generation using GANs/VAEs. - Differences in data quality and collection methods across institutions remain a challenge.
Ethical and regulatory concerns	- Privacy concerns with large-scale data use risk violating laws like Health Insurance Portability and Accountability Act (HIPAA) and General Data Protection Regulation (GDPR). - Biases in training data may lead to disparities in diagnosis across demographics. - Federated learning, encryption, and anonymization enhance privacy compliance. - Institutions conduct bias audits to address underrepresentation and ensure equitable performance.
Model interpretability	- DL models’ "black box" nature makes it difficult to explain predictions. - Lack of interpretability affects trust, clinical adoption, and patient safety. - Techniques like Grad-CAM, SHAP, and LIME improve transparency by highlighting influential features or regions in predictions. - Despite advancements in explainability, risks of bias and errors persist.
Deployment and scalability	- High computational demands (graphics processing units/tensor processing units) and infrastructure disparities hinder scalability, especially in low-resource settings. - Exacerbates socioeconomic gap between communities of different standings. - Poor integration with clinical systems (EHR, PACS) disrupts workflows and reduces adoption. - Cloud-based deployment offers scalable solutions but raises concerns about data security and privacy. - Standards like DICOM and FHIR are critical for seamless data exchange.
Clinical trust	- Concerns about accuracy, reliability, and lack of interpretability. - AI may flag genetic variants without known clinical relevance, making it hard for clinicians to act on such results confidently. - Unvalidated AI outputs can lead to false positives or misinterpretations, potentially resulting in decisions that deviate from clinical guidelines. - XAI is being developed to make AI decisions more transparent, helping clinicians understand the reasoning behind model predictions. - For AI to be trusted and adopted in clinical settings, thorough validation, interdisciplinary collaboration, and regulatory supervision are essential.

### Current Clinical Programs

#### Current Clinical Trials

Current clinical trials in DL for cancer detection represent a pivotal step in integrating AI into precision oncology. These trials aim to validate the efficacy, safety, and real-world applicability of DL models across various cancer types and diagnostic modalities. For instance, the DENSE-Lung Study focuses on leveraging DL algorithms to analyze low-dose CT scans for early lung cancer detection, aiming to minimize false positives and missed diagnoses.^81^ Similarly, the AI identifying polyps in a real-world colonoscopy trial applies AI-assisted diagnosis systems to identify and classify lesions in real-time from images of the colon.^82^ Prostate cancer detection is being explored through the AI and radiologists in the prostate cancer detection in MRI trial. This study aims to evaluate the effectiveness of AI algorithms in enhancing the diagnostic accuracy and efficiency of prostate MRI for detecting prostate cancer.^83^ Furthermore, the development of the Alu profile learning using sequencing ML approach highlights the application of DL algorithms in analyzing liquid biopsy data for early cancer detection and monitoring. By leveraging ML to identify tumor-specific DNA mutations and methylation patterns from blood samples, this approach offers a minimally invasive and highly sensitive method for diagnosing cancers.^84^ Notably, DermaSensor, after 3 clinical trials (DERM-SUCCESS Prospective Skin Cancer Validation Study, DERM-ASSESS III Prospective Melanoma Validation Study, and DERM-SUCCESS Prospective Clinical Utility Study), has demonstrated that it can classify skin lesions with accuracy comparable with dermatologists. This model, trained on a vast data set of skin lesion images, has been integrated into various clinical tools for the early detection and diagnosis of cancer. Its involvement in clinical trials aims to validate its effectiveness in real-world applications, enhancing diagnostic precision and accessibility.^85^^,^^86^ Furthermore, in the field of pathology, Paige.AI is conducting clinical trials to validate AI-assisted cancer detection in pathology. Their model analyzes WSIs to identify tumor subtypes and molecular biomarkers.^87^

#### Current Clinical Applications

Current clinical applications of DL in cancer detection have significantly enhanced diagnostic workflows, offering clinicians advanced tools for improved accuracy and efficiency in early detection. In breast cancer detection, Google Health’s Mammography AI demonstrated superior performance compared with radiologists in detecting malignancies during mammography screening, as validated in a study highlighting its effectiveness.^88^ Furthermore, Transpara by ScreenPoint Medical is an AI-based system that utilizes DL to detect suspicious regions in 2D/3D mammograms, aiding in breast cancer detection. It provides radiologists with a supportive tool to enhance diagnostic accuracy, particularly in cases involving dense breast tissue. Clinical validations demonstrate its potential to improve early cancer detection and reduce recall rates.^89^ For lung cancer, Zebra Medical Vision’s AI has been deployed to identify lung nodules in CT scans, with research emphasizing its accuracy in large-scale screening programs.^90^ For dermatology, applications like SkinVision have utilized CNNs to enhance melanoma detection, achieving high diagnostic accuracy and sensitivity compared with dermatologists.^90^ These active clinical applications exemplify how DL is transforming cancer diagnostics, paving the way for more precise, timely, and personalized care. As mentioned above, DL models such as ResNet50 and Hover-net are being used to analyze histopathological images of breast cancer. These models aid in classifying tumor subtypes and predicting metastasis risk, thereby enhancing the diagnostic accuracy of cancer subtypes.^43^ Current clinical programs for DL in this field are summarized in Table 4.

**Table 4:** Current Clinical Programs

Clinical programs	Key points
Current clinical trials	- *DENSE-Lung Study*: Uses DL for low-dose CT scan analysis to enhance early lung cancer detection by reducing false positives and missed diagnoses. - *AI in Colonoscopy*: Real-time lesion identification and classification during colonoscopies. - *AI in Prostate MRI*: Evaluates DL in improving diagnostic accuracy for prostate cancer detection in MRIs. - *A-PLUS*: A ML approach analyzing liquid biopsy data for detecting tumor-specific DNA mutations and methylation patterns. - *DermaSensor*: Validated through multiple trials, this DL model achieves dermatologist-level accuracy in classifying skin lesions, now integrated into clinical tools for early skin cancer detection. - Paige AI: analyses WSIs to identify tumor subtypes and molecular biomarkers.
Current clinical applications	- *Breast Cancer*: - Google Health's Mammography AI: Outperforms radiologists in detecting malignancies during mammography screenings. - *Transpara*: AI tool for identifying suspicious regions in 2D/3D mammograms, especially useful in dense breast tissue cases. - *Lung Cancer*: Zebra Medical Vision’s AI identifies lung nodules in CT scans, proving effective in large-scale screening. - *Dermatology*: SkinVision applies CNNs for melanoma detection with accuracy comparable to dermatologists. - ResNet50 and Hover-Net: classify tumor subtypes and predict metastasis risk, improving diagnostic accuracy of cancer subtypes.

### Future Directions of DL in Cancer Detection and Diagnosis

#### Expanding Collaborative Research

Expanding collaborative research in cancer detection using DL between AI researchers, oncologists, ethicists, and policymakers can drive innovation, improve generalizability, and ensure the ethical and scalable deployment of AI tools in diverse health care settings.^91^ Initiatives that foster data sharing while maintaining patient privacy will accelerate progress.^92^ Researchers could create multinational consortia to pool anonymized data for training and validating DL models, ensuring representation across demographics and regions.^93^ Furthermore, the world should adopt standardized data formats and protocols to enable seamless sharing and integration across institutions.^94^ To ensure DL’s robustness and reliability, hospitals and health care systems should conduct collaborative trials to validate DL models.^91^ Health care institutions should also create platforms where researchers can test their models against standardized data sets, promoting transparency and comparability.^95^

#### Exploring Next-Generation AI Techniques

Exploring next-generation AI techniques is crucial for advancing cancer detection using DL. Emerging technologies and methodologies promise to overcome existing limitations, enhance diagnostic precision, and revolutionize the field of oncology. Emerging techniques such as reinforcement learning and federated learning hold promise for enhancing model robustness and scalability.^96^ Additionally, synthetic data generation using GANs can address data limitations by augmenting existing data sets.^97^ New technologies and systems have a large potential for filling in gaps in current methods, allowing for faster detection of cancer and more timely treatment.

#### Transformers

Transformers, originally developed for natural language processing, are being adapted for image and multimodal data analysis.^98^ Hierarchical transformers are an advanced adaptation of the transformer architecture that excels at analyzing multiscale data. This approach is particularly significant in cancer detection, where understanding both fine-grained (eg, cell-level) and coarse-grained (eg, tissue-level) features is critical. These models process data at varying levels of granularity, integrating local and global information to provide a more comprehensive understanding of tumor characteristics.^99^ As this is a relatively new concept, more work could be done on real-world validation. Few hierarchical transformer models have been validated in real-world clinical workflows.^100^ Thus, conducting large-scale clinical trials to test model performance in diverse health care settings and assessing models for diagnostic accuracy across different patient populations, scalability in busy clinical environments, and integration into radiology, pathology, and oncology workflows would drive transformative advancements.

#### Interpreting Multiomics Studies With DL

DL is revolutionizing the use of multiomics studies for cancer detection by enabling the integration and analysis of diverse biological data, such as genomics, transcriptomics, proteomics, epigenomics, and metabolomics, to uncover complex patterns that traditional methods often miss. With the integration of DL models, a more comprehensive overview of multiomics data sets can be obtained, as they can bypass human limitations. Researchers are developing innovative DL frameworks to address the challenges of heterogeneity and high dimensionality in multiomics data. For instance, DeepMO, a variational autoencoder-based pipeline that learns common latent representations across data types by using TCGA data set, can achieve superior performance in cancer subtype classification.^101^ In addition to TCGA data set, other data sets such as the genotype-tissue expression project and ArrayExpress can also provide different services in cancer detection for DL in multiomics. One major service is the profiler of multiomic data. Profiler of multiomic data is a bioinformatics visualization tool. It is a standalone, interactive software designed for analyzing large genomic cancer data sets along with their associated clinical information. The Profiler of Multi-Omic data (PROMO) offers functionalities such as survival analysis, expression heatmaps, volcano plots, and correlation matrixes, facilitating the exploration of molecular patterns in cancer using multiomics data.^102^ Another service is the gene expression profiling interactive analysis 2, a web-based platform that analyzes RNA sequencing expression data from TCGA and the genotype-tissue expression project, and is used for comparing gene expression between cancerous and normal tissues. Both tools help researchers prepare and understand multiomics data sets, which can then be used to train DL models that predict cancer risk, type, or treatment response more accurately.^103^

Similarly, graph NN, such as GraphOmics, model interactions between biological entities, including genes and proteins, to enhance biomarker discovery, as demonstrated in studies on pancreatic cancer.^104^ Attention mechanisms, such as those used in TransOmicsNet, dynamically prioritize important features across omics layers, identifying key drivers of diseases like glioblastoma.^105^ Time-series models, such as TimeOmics, analyze longitudinal data to track cancer progression and predict treatment outcomes, as seen in prostate cancer studies.^106^ XAI tools, such as XOmiVAE, make DL models interpretable by identifying and ranking critical omics features, including the contribution of each gene and latent dimension to each classification prediction, thereby aiding trust and adoption.^107^ Generative models, such as cGANomics, augment data sets by simulating synthetic omics data, particularly for rare cancer types.^108^ Cloud-based platforms, such as DNAnexus, democratize access to these tools, enabling researchers to accelerate biomarker discovery and diagnostic workflows.^109^ Emerging trends include single-cell multiomics DL models for precise cancer subtyping,^110^ federated learning to train models securely across institutions without sharing sensitive data,^111^ and real-time multiomics integration for on-the-fly diagnostics.^112^These advancements highlight how DL is opening possibilities for researchers to gain access to a more well-rounded and insightful view of multiomics studies, providing unprecedented opportunities for early cancer detection and personalized treatment strategies, even for those unfamiliar with the complexities of omics data. However, the integration of DL still requires researchers to convert the data into other forms that can be analyzed by the algorithm. For example, numerical molecular data cannot be easily read by DL and has to be manually transformed into different forms. Furthermore, different omics fields employ distinct data collection methods, resulting in various data types, ranging from binary to more complex oscillatory formats. Therefore, this limitation hinders the tracking and analysis of data for cancer detection.

Some fusion models that are present today include pathomic fusion. It combines histopathology, in the form of WSIs, with genomics and transcriptomics. This fusion method primarily targets Giloma and utilizes a late-fusion DNN. It integrates features from WSIs with gene expression and genomic mutations and has, to date, achieved state-of-the-art survival prediction and subtype classification.^41^ Another fusion model is called the MultiSurv model. This model fuses clinical data, mRNA expression, DNA methylation, copy number variation, and WSIs. It primarily targets Pan-cancers across 33 TCGA types and utilizes a deep survival model with an early late hybrid fusion. It predicts patient survival by using attention-based fusion of multiomics and learns modality-specific features, aligning them to a joint latent space.^113^

Molecular data present complexities and pose challenges to cancer detection, especially in multiomics, with major challenges including the presence of high-dimensional data, gene redundancy, and the fact that different omics types behave differently. For dimensionality problems, one can use the autoencoder-assisted graph convolutional neural network, which combines autoencoders with GCNs to reduce dimensions and learn relationships in gene expression data. It has already been used in pan-cancer classification tasks with TCGA data.^114^ For gene redundancy, we have the Boruta Algorithm. It is A random forest-based method that identifies all relevant genes while removing redundant or irrelevant ones, usually applied in transcriptomic-based breast cancer classification.^115^ To combat this, the MultiSurv algorithm utilizes dedicated submodels to establish feature representations of clinical imaging and various high-dimensional omics data modalities. This can effectively tackle different types of data one at a time, reducing the chances of data incompatibility.^113^

#### Solving Challenges

Beyond simple rotations and flips, future work could involve domain-specific augmentations, such as simulating variations in histopathology staining and synthetic tumor growth patterns. This can be used to solve data set size limitations. To address standardization issues, we propose creating international standardized protocols for medical image acquisition in areas such as resolution, staining methods, and magnification, thereby minimizing data variability and harmonizing data sets globally. In cases where discrepancies are inevitable, we propose that institutions develop and adopt federated learning protocols with federated domain adaptation, enabling models to handle slight data set distribution shifts between institutions. For smaller institutions, we propose using a federated transfer learning algorithm to assist smaller institutions with fewer samples. Furthermore, Edge AI models can also be developed for use in rural hospitals, along with lightweight architectures optimized for health care, similar to MobileNet variants.^116^ For ethical concerns, we believe that the World Health Organization Ethics and Governance of AI for Health does a commendable job addressing the ethics aspect of the challenge; however, it can be further improved by instituting mandatory actions, rather than just strongly encouraging institutions. Widespread homomorphic encryption and secure multiparty computation should also be enforced to ensure the privacy of data transferred. To reassure patients and restore clinical trust, we propose introducing confidence thresholds that flag ambiguous cases for additional human review. Furthermore, models should be accompanied by detailed “model cards” explaining their training data, limitations, failure modes, and tested demographic performance. This and other future directions are summarized in Table 5.

**Table 5:** Future Directions of DL in Cancer Detection and Diagnosis

Future direction	Key points
Expanding collaborative research	*Importance of Collaboration*: Cross-disciplinary efforts between AI researchers, clinicians, ethicists, and policymakers are essential for innovation and ethical scalability in DL-based cancer detection. *Data Sharing Initiatives*: Developing international consortia to share anonymized, standardized datasets ensures diverse and representative data for training robust models. *Standardization*: Adopting universal data formats and protocols enhances compatibility and integration across institutions. *Collaborative Validation*: Conducting multinational trials and creating platforms for testing against standardized datasets ensures transparency, comparability, and real-world reliability.
Exploring next-generation AI techniques	*Reinforcement Learning*: Useful for optimizing decision making in clinical workflows, improving diagnostic accuracy, and personalizing patient management. *Federated Learning*: Promotes secure, decentralized model training across institutions, addressing privacy concerns and enabling access to larger datasets. *Synthetic Data Generation*: GANs and similar tools augment datasets, addressing limitations in rare cancer types and improving model performance.
Transformers in cancer detection	*Application of Transformers*: Initially developed fornatural language processing, transformers are now applied to image and multimodal data analysis in oncology. *Hierarchical Transformers*: Analyze multiscale data by integrating fine-grained (e.g., cellular) and coarse-grained (e.g., tissue-level) features for improved tumor characterization. *Future Directions*: Conducting large-scale trials for real-world validation in diverse settings, assessing scalability, and integrating transformers into radiology, pathology, and oncology workflows.
Interpreting multiomics studies with DL	*DL Integration in MultiOmics*: Facilitates the analysis of diverse biological datasets (genomics, proteomics, metabolomics, etc.), uncovering complex patterns traditional methods miss. *Innovative Frameworks*: • *DeepMO*: Variational autoencoders unify data modalities for cancer subtype classification (e.g., TCGA dataset). • *Graph Neural Networks*: Model interactions between genes and proteins, enhancing biomarker discovery (e.g., pancreatic cancer). • *TransOmicsNet*: Attention mechanisms dynamically prioritize features across omics layers for disease insights (e.g., glioblastoma). • *Time-Series Models*: TimeOmics tracks cancer progression and predicts treatment outcomes (e.g., prostate cancer). • *Explainable AI (XAI)*: Tools like XOmiVAE make DL interpretable, identifying critical features for classification predictions, fostering clinician trust. • *Generative Models*: cGANomics generates synthetic omics data, aiding in rare cancer studies. *Emerging Trends*: Single-cell multiomics models, federated learning for secure collaborations, and real-time multiomics integration for rapid diagnostics. *Fusion Models:* 1. *Pathomic Fusion*: Integrates features from whole-slide images with gene expression and genomic mutations 2. *MultiSurv*: redicts patient survival using attention-based fusion of multiple omics, and learns modality-specific features and aligns them to a joint latent space. Also uses dedicated submodels to establish feature representations of clinical imaging. *AE-GCN*: combines autoencoders with GCNs to reduce dimensions and learn relationships in gene expression data. Boruta Algorithm: Random forest-based method that identifies all relevant genes while removing redundant or irrelevant ones
Solving challenges mentioned	- Domain-specific augmentations - Create international standardized protocols for medical image acquisition in areas such as resolution, staining methods, magnification - Develop and adopt federated learning protocols with federated domain adaptation - Federated transfer learning algorithm for smaller institutions - Edge AI models for rural areas - Instituting mandated actions in the WHO Ethics and Governance of AI for Health - Widespread homomorphic encryption and secure multiparty computation - Introduce confidence thresholds - Model Cards accompanying every AI model

## Conclusion

Overall, DL has revolutionized cancer detection and diagnosis by offering unparalleled advancements in imaging-based diagnostics, genomic analysis, and multimodal data integration. DL excels in early cancer detection by identifying complex patterns in imaging and genomic data, thus enhancing diagnostic precision. However, its full potential is hindered by challenges such as data limitations, ethical concerns, interpretability issues, and deployment barriers in clinical settings.

Researchers should prioritize developing next-generation AI techniques, including XAI to enhance model transparency and trust, as well as federated learning to enable collaborative training while preserving privacy. Tools like synthetic data generation via GANs and VAEs should be leveraged to overcome data scarcity, particularly for rare cancers. Integrating multimodal data, including genomic, imaging, and clinical records, is crucial for creating holistic and personalized cancer diagnostic systems.

Clinicians play a pivotal role in the codevelopment and effective utilization of AI tools. Standardizing data input methods, adhering to formats like DICOM and FHIR, and capturing comprehensive, structured clinical notes are essential steps to enhance AI training and performance. Clinicians should also ensure the accuracy and diversity of data to reduce biases and improve model generalizability. Furthermore, interdisciplinary collaborations with AI researchers can help validate models, refine workflows, and align tools with real-world clinical needs. Training programs to familiarize clinicians with interpreting AI outputs will be critical for seamless adoption and integration into practice.

By addressing these challenges and fostering a collaborative ecosystem, DL can continue to redefine cancer diagnostics, enabling earlier detection, equitable access, and improved patient outcomes. Future advancements in AI, combined with responsible deployment and clinician involvement, will enable these technologies to realize their transformative potential in oncology. Future research should focus on real-world validation of transformers, testing model performance in diverse health care settings, and assessing models for diagnostic accuracy across different patient populations, as well as scalability in busy clinical environments and integration into radiology, pathology, and oncology workflows.

## Potential Competing Interests

The authors report no competing interests.

## Declaration of Generative AI and AI-Assisted Technologies in the Writing Process

During the preparation of this work the authors used ChatGPT-4, an AI language model developed by OpenAI in order to refine language and summarize the text. After using this tool, the authors reviewed and edited the content as needed and takes full responsibility for the content of the publication.
