# Supplementary material for: Deep Learning Applications in Clinical Cancer Detection: A Review of Implementation Challenges and Solutions
Source: Mayo Clin Proc Digit Health. 2025 Jul 18;3(3):100253. doi: 10.1016/j.mcpdig.2025.100253 (PMC12351333; doi:10.1016/j.mcpdig.2025.100253)
Supplement: Supplemental Material [file mmc1.docx]

**Supplemental Appendix**

**Flow chart of studies using PRISMA guidelines**

| Items | Specification |
| --- | --- |
| Date of search | The first search was conducted on 11/28/24. The last search was conducted on 4/18/25. |
| Databases and other sources searched | The databases searched were PubMed and IEEE Xplore |
| Search terms used | 1. Boolean Logic: connecting words like “AND”, “OR” to narrow down or widen up the scope of the search. 2. Truncation: we searched for terms that began with a specific string by enclosing the key term in double inverted commas (“”) at the end of a root word. The word strings used in the search were “Deep Learning” or “Artificial Intelligence” AND "Cancer detection". |
| Timeframe | 2018-2024 |
| Inclusion and exclusion criteria | Inclusion criteria: age greater than 18 years old; DL novel models; peer & non-peer reviewed publications; cancer  Exclusion criteria: non-cancer. |
| Selection process | Using the search criteria, 3392 articles were found. Two independent reviewers (D.M.) and (Y.Z.L.) selected articles that fit the inclusion criteria. 145 papers met the study eligibility criteria out of 3392. A third reviewer (W.Y.K.) served as a tiebreaker to resolve disagreements. |
